# Conjectures, refutations and the search for truths: Science, symbolic truths and the devil

**DOI:** 10.15252/embr.201949924

**Published:** 2020-01-13

**Authors:** G Paolo Dotto

**Affiliations:** ^1^ University of Lausanne Epalinges Switzerland; ^2^ Massachusetts General Hospital Boston MA USA; ^3^ International Cancer Prevention Institute Epalinges Switzerland

**Keywords:** S&S: History & Philosophy of Science

## Abstract

In times of fake news, post‐truths and post‐science, the principles of science can inform all kinds of inquiries into the true nature of reality.

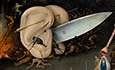

What is truth? This is the question Pilate asks Jesus at the beginning of Bulgakov's^†^ novel *The Master and Margherita*. In his conversation with a newspaper editor and convinced atheist, the devil, in the person of Professor Woland, supports the veracity of his account of Jesus’ trial with the startling prediction that the good editor will soon be dead: His head will be chopped off as the consequence of the action of an unsuspecting housewife. This turns out to be true: The man slipped on cooking oil spilled on the ground and falls under the wheels of a streetcar that severs his head.

## A time of post‐truths?

Enlightened citizens of the 21^st^ century now dismiss all talk of the devil as superstitions of medieval people locked in their self‐incurred tutelage. But what is the possibility that the devil, having donned all personalized forms of identification—Satan, Lucifer, Woland, Mephisto and so on—is still around and seduces us to commit evil deeds? Or viewed from another perspective: Is the present time of confusion and “post‐truths” related to a loss of “objective” truths on which we base rational decisions? It seems that anything is “true” these days amidst exponentially increasing complexities and conflicting messages. However, despite recent claims of “post‐science”, the principles of scientific inquiry, if correctly understood and applied, can help to clarify and reaffirm the reality on which we are all grounded. It is on this reality that we depend to agree with others about basic facts and to move on.

But what is the possibility that the devil, having donned all personalized forms of identification […] is still around and seduces us to commit evil deeds?

The philosopher Karl Jaspers stated that *ontology* and *peri‐echo‐ontology* are the “science” of the encompassing *being* that sustains and drives existence. These sound like arcane and obscure words that only philosophers or theologians care about. However, *being* is, above all, an essential element of language. It is only by declining the verb *to be* that we can think and express who and where we *are* in connection with others. As such, we cannot get out of *being*, as it is on *being* that we depend in life. And it is for that reason that we have to understand *being* and the world in which we are.

The truths to which philosophy and ethics aspire should not be confused with scientific discoveries. Nonetheless, all forms of knowledge are only approximations of the reality in which we are immersed and in which we need to orient ourselves. As such, scientific and philosophical/ethical investigations have a single common basis, and there is a need of communication and mutual understanding to firm our steps and avoid shifting and ultimately destroying paradigms. This is nothing new. Socrates claimed to know only one thing, that he knew nothing. And yet, he lived—and died—for his uncompromising search of truth. As quoted by Hannah Arendt in the first chapter of *The Origins of Totalitarianism*, Plato in his fight against the Sophists of his time pointed out the insecure position of truth in the world, since “from opinions comes persuasion and not from truth” (*Phaedrus*, 260).

## Looking for truth

The main impetus for scientific investigations—and philosophical and ethical inquiries—is “simply” to seek and tell the truth. It is not a vague, nebulous truth, based on ill‐defined notions and personal feelings; it is a truth that provides us with directions and that helps us to understand where we are, what is hot or cold, black or white, right or wrong.

The main impetus for scientific investigations – and philosophical and ethical inquiries – is ‘simply’ to seek and tell the truth.

And yet, any truth is never final. It is based on gathering information and formulating working hypotheses that need to be validated or refuted by hard evidence, starting from and delving back into the “reality” into which we are all immersed. A simple fact—which is unappreciated by those on the outside—is that a single scientific paper, < 10 pages long and with as few as 3–4 figures, is the result of several years of work by a team of people who dedicate enormous amounts of time and efforts along with a serious financial commitment. Their conclusions are subjected to rigorous testing and review before they can be published to serve as a premise for the work of others.

But it is also important to keep in mind that behind any scientific article, no matter how well and carefully documented, can be strong biases, just like behind any philosophical work. Scientists and non‐scientists alike have to deal and operate with their individual points of view, history and motivations.

## Principles of scientific inquiry

With these limitations in mind, a few principles of scientific inquiry can be formulated which would also help philosophical and ethical investigations.

**Figure 1:**
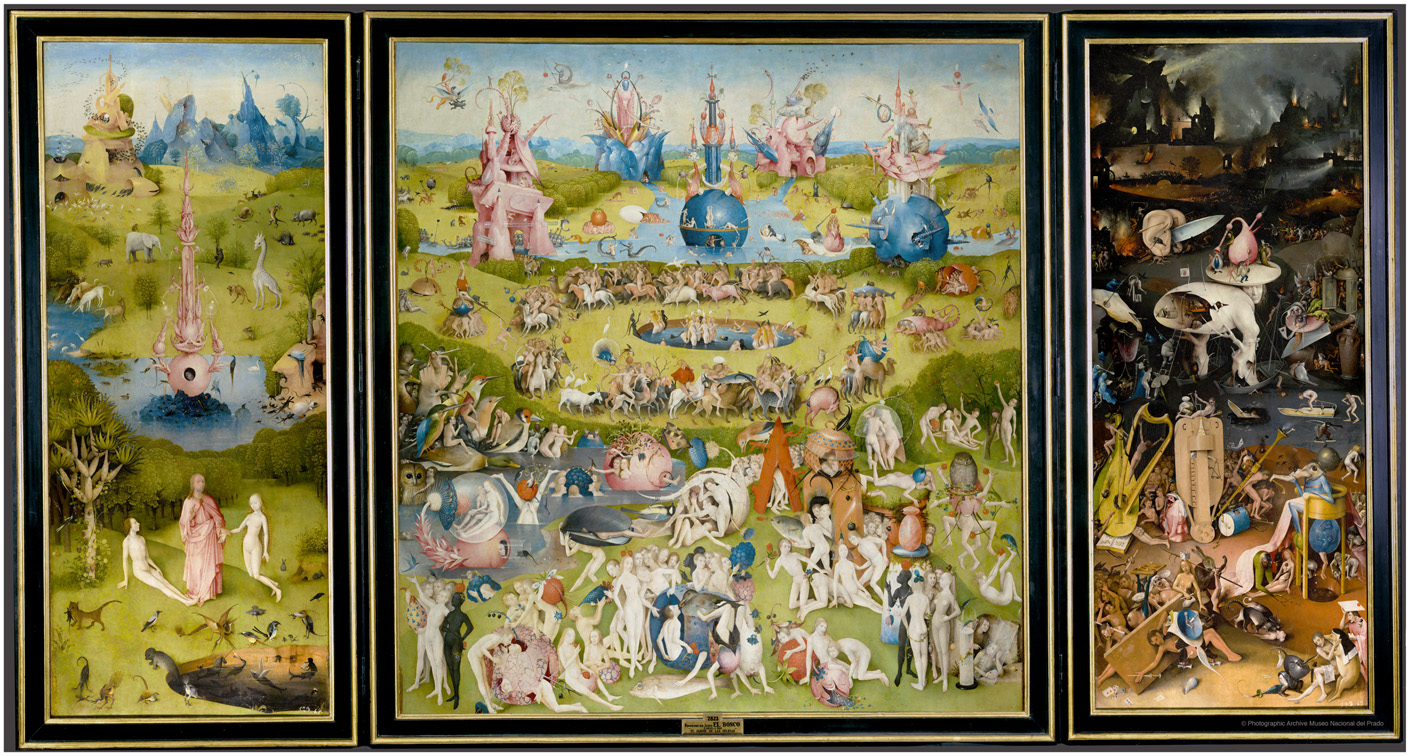
“The Garden of Earthly Delights” (1490–1510) by Hieronymus Bosch Museo Nacional del Prado, Madrid, Spain. © Photographic Archive Museo Nacional del Prado.

*A first principle* of scientific inquiry is epistemological: Scientific truth can only be approached by approximation, through the process that Karl Popper called “*Conjectures and refutations”*. When this principle is forgotten and science becomes “dogmatic”, it negates itself.
*A second principle* is that there is a direction in discovery. Even if only tentative and “hypothetical”, scientific truth does not allow to go back, it is only possible to move forward. As such, scientific discoveries have a relative value but are never arbitrary: They serve as stepping stones on which to build a house.
*A third principle* is that scientific truths can only be “symbolic”. The etymological meaning of *symbol* is “putting together” (from the Greek *syn‐ballein*); it refers to a multidimensional reality and is capable of synthesizing complexity into manageable simplicity. The value of a symbolic word is greater than the word itself and is part of the reality to which it points (Fig 1). At the same time, symbols need to be carefully defined; otherwise, symbolic language becomes babble.

## The power of symbols

The symbols of mathematics and physics, starting from numbers, are fundamental for these disciplines, as for all other advances in science to which they have led. In his book “Introduction to Mathematical Philosophy”, Bertrand Russell expressed great admiration for Giuseppe Peano, an Italian mathematician, who laid the foundation of mathematical logic starting with his first axiom that zero is a natural number (https://en.wikipedia.org/wiki/Peano_axioms). The notations he introduced are just symbols, but it is from these symbols that the great works of Kurt Gödel and John von Neumann arose which led to Boolean language and computers. In biology and medicine, words such as *genetics*,* epigenetics* and *metabolomics* are equally important symbolic terms referring to dynamic and complex realities that determine our lives. The most sophisticated symbols in the natural sciences are “theories”: formulations of principles that provide a keystone for interpretation, prediction and more research.

*Evolution* is a theory of theories that provides a basis for deciphering biological phenomena in quantitative terms. According to the British historian Peter Watson, the theory of evolution was the most important intellectual and conceptual achievement of the 19^th^ century as it provides an interpretative key for all of the living reality of which we are part. Yet, it must be emphasized that the theory of evolution, like any other theory, is a “symbolic expression of facts”, that, as any scientific truth, must be tested and challenged by “Conjectures and Refutations”. Even long‐held views must always be reconsidered, challenged and even discarded as part of science's forward process. Scientists should explain to others the dynamic process by which these results are reached.

**…** the theory of evolution, like any other theory, is a “symbolic expression of facts”, that, as any scientific truth, must be tested and challenged by ‘Conjectures and Refutations’.

Like science, philosophy is symbolic. The deeper philosophical inquiries delve into reality, the more difficult the language becomes. Of necessity, “going beyond” realities immediately connected with experience implies the use of words with a different meaning from common use. Precise definitions become therefore essential for mutual understanding, and many debates result from problems of communication, rather than real disagreements. On the other hand, faced with the mystery of ultimate realities, the language of philosophy must be as concise as possible. Like scientific truths, philosophical and ethical truths can be expressed in symbolic words: *natural law* and *sacrality of life*, or *the rights of man*, are all symbols that point “beyond”.

To have an impact on reality, symbols must be clear‐cut and well defined. At the same time, their validity must be guaranteed, in science as in all other kinds of human activity, by a recognized “authority”—the “contribution to the common good” ceases to be an abstract concept as soon as the tax revenue authority demands its share. In today's society, all forms of authority seem to be fading away, which carries the risk that symbolic truths, on which these authorities are based, fade with them.

Throughout history, religions have had the fundamental task of expressing ontological and ethical truths with the authority of “symbolic dogmas”. The myth of Isis and Osiris or the Song of Songs gave voice to the mysterious power of life and death. “In the beginning was the Word, and the Word was with God, and the Word was God”. The first verse of John's Gospel describes how a single word became flesh. It has brought together God and Man at a precise time in history, not as an abstract concept but a transforming reality, which has been personally encountered by many, like Saul on his way to Damascus.

## The devil in confusion

When symbols lose their grasp, they are ultimately discarded. To many people, the existence of the devil, like that of God, seems like a thing from a medieval past. The devil's various embodiments, such as the one Faust was dealing with, seem only a product of fiction or imagination. They gave tangible *personam* to the existential and universal condition by which even now we are threatened.

What then is the devil? If the etymological meaning of “symbol” is to put together, that of “devil” is the opposite, to divide: The old English word *deofol* derives from the Latin *dia‐bolus* and the Greek *dia‐ballein*.

As discussed above, any form of truth is expressed by symbols. Each symbol, in turn, is based on other symbols, concepts and words. One can say that a symbol is as a building made of bricks, which must be properly squared to erect a stable construction. If the bricks are not squared, they do not fit well together and eventually the entire edifice crumbles.

S*ym‐bolus*, as a construction, carries therefore within itself the seeds of its own destruction and can turn into a *dia‐bolus*. The devil can be found wherever there is confusion, when symbols lose their meaning and the words or concepts on which they are based are ill interpreted or manipulated. The white can be mistaken for black or both become a mixture of greys, a fog that blocks the view.

There is another venue for the devil to affirm its power. Each building risks becoming a fortress, excluding those outside and imprisoning those inside. Symbolic constructions can become thus an obstacle to mutual communication and understanding. It can take a huge amount of energy and great personal risk to force open a building's doors. The exponential increase of knowledge and complexities in any individual field is such that it is much easier and comfortable to build barriers and walls than to pull them down.

The existence of the devil is therefore not simply a superstition of ancient times, or an abstract concept of little importance. The devil, as a principle of confusion and discord, is wherever there is lack of clarity and fear of opening up to others. The devil is very much present and forceful whenever there is ignorance and superstition, but also when any group of people feel justified to impose their own truths or values on others.

The devil is very much present and forceful whenever there is ignorance and superstition, but also when any group of people feel justified to impose their own truths or values on others.

## Exorcism

The principles of science can come to the rescue and serve as a solid point of reference to address difficult ontological and ethical problems. Falsification or misrepresentation of truths of any kind is a great threat to all of us. At the same time, is the fallacy of all divisions between “us and them”, be it ethnicity and race, sex, political party or creed.

Scientists can play an important role in this difficult moment, provided they escape the devil's temptation of viewing “their truths” as the unquestionable foundation of all reality. Scientists need to open doors and windows of their well‐constructed building and explain their views and speak with others, so that we all can work together in *a symphonic world*. It is worth trying, as the stakes are high.

## Footnotes

^†^ Correction added on 5 February 2020, after first online publication: Nabokov was corrected to Bulgakov.

